# The Assembly of Diverse Immune Receptors Is Focused on a Polar Membrane-Embedded Interaction Site

**DOI:** 10.1371/journal.pbio.0040142

**Published:** 2006-04-25

**Authors:** Jianwen Feng, Matthew E Call, Kai W Wucherpfennig

**Affiliations:** **1**Department of Cancer Immunology and AIDS, Dana-Farber Cancer Institute, Boston, Massachusetts, United States of America; **2**Program in Immunology, Harvard Medical School, Boston, Massachusetts, United States of America; **3**Department of Neurology, Harvard Medical School, Boston, Massachusetts, United States of America; National Jewish Medical and Research Center/Howard Hughes Medical InstituteUnited States of America

## Abstract

The majority of receptors responsible for activation of distinct cell types within the immune system assemble with dimeric signaling modules through interaction of a basic transmembrane residue with a pair of acidic residues of the signaling dimer. Because assembly of other membrane proteins requires specific interactions along extended stretches of transmembrane helices, we examined how transmembrane sequences flanking the polar interaction site contribute to assembly for three receptors that associate with different signaling modules—the natural killer cell receptors KIR and NKG2D and the Fc receptor for IgA, FcαRI. The KIR and NKG2D receptors assembled with the DAP12 and DAP10 dimers, respectively, even when the entire KIR or NKG2D transmembrane domains were replaced by polyleucine sequences with a properly positioned basic residue. In contrast, a high degree of specificity for the basic side chain could be observed because the KIR–DAP12 and FcαRI–Fcγ interactions favored lysine or arginine, respectively. Steric hindrance among incompatible extra-membranous domains and competition for signaling modules also contributed to specificity of assembly. These results demonstrate that these interactions are focused on the polar site created by three ionizable transmembrane residues, and explain how the DAP12 and Fcγ signaling modules can assemble with large, non-overlapping sets of receptors that have highly divergent transmembrane sequences.

## Abstract 2

By mutating all residues but one to polyleucine in diverse immune receptors, the authors find that only one polar interaction embedded in the membrane is required for their specificity and assembly.

## Introduction

The activation of cells in the immune system is controlled by a large and diverse group of surface receptors that belong to two distinct protein families, the immunoglobulin and the C-type lectin families. The ligand-binding subunits of these receptors have only short cytoplasmic domains and activation signals are instead transduced through associated dimeric signaling modules whose cytoplasmic domains are phosphorylated at tyrosine residues following receptor ligation. Important examples include the T cell receptor (TCR) that triggers activation following recognition of viral and bacterial peptides bound to major histocompatibility complex molecules; the KIR, NKG2D, and NKG2C/CD94 receptors that control natural killer (NK) cell–mediated lysis of transformed or infected cells; and several Fc receptors, including the FcαRI receptor for IgA, that are responsible for receptor-mediated uptake of antibody-decorated pathogens [ 1– 6].

We previously showed that assembly of the TCR with its three signaling modules (CD3δɛ, CD3γɛ, and ζζ) is organized by specific interactions among ionizable transmembrane (TM) residues [ 7]. Each of these assembly steps involves a particular basic TCR TM residue and a pair of acidic TM residues of the interacting signaling module. Formation of the appropriate receptor structure is therefore dependent on proper placement of a total of three basic and six acidic TM residues. The requirement for both acidic residues at each interaction site was shown by mutagenesis experiments in which substitution of either one of the two acidic residues led to an assembly defect [ 7]. More recently, we showed that the same structural arrangement among one basic and two acidic TM residues guides the assembly of a variety of activating receptors in the immune system, including members of the immunoglobulin (KIR, FcαRI) and C-type lectin (NKG2C/CD94, NKG2D) families [ 8, 9]. The membrane-localized assembly mechanism is therefore relevant for activating receptors expressed by many different cell types of hematopoietic origin.

The majority of activating receptors assemble with the DAP10, DAP12, or Fcγ signaling modules that form disulfide-linked homodimers with short extracellular domains. Their cytoplasmic domains carry characteristic tyrosine-based phosphorylation motifs, either the classical immunoreceptor tyrosine-based activation motif that recruits ZAP-70 or Syk tyrosine kinases in its phosphorylated state (DAP12, Fcγ, and TCR-associated signaling modules) or the YxxM motif that binds the p85 subunit of PI-3 kinase (DAP10) [ 10– 13]. The TM aspartic acid pair of these signaling dimers is located either close to the center (DAP10 and DAP12 dimers) or in the upper third of the TM domains (Fcγ dimer), matching the location of the basic TM residue of the interacting receptors. The DAP10 dimer specifically associates with the NKG2D receptor that triggers NK cell–mediated lysis of infected or transformed cells through recognition of stress-regulated surface molecules [ 12]. The closely related DAP12 dimer (also termed KARAP) forms the signaling component of a large group of activating receptors, such as the KIR and NKG2C/CD94 receptors expressed by NK cells, as well as a family of receptors (including TREM-1, TREM-2, PILRβ, SIRPβ1, and IREM-2) expressed in different patterns by subpopulations of myeloid cells (monocytes, dendritic cells, microglia, neutrophils, and osteoclasts) [ 10, 13– 15]. The Fcγ signaling module has strong sequence homology to the TCR ζ chain [ 16, 17] and serves as a signaling component for several Fc receptors and a variety of other receptors [ 5, 11, 18].

The experiments conducted so far clearly demonstrate that the TM domains are sufficient to mediate assembly and that the ionizable TM residues play a critical role. However, the contribution of the remaining TM sequences is largely unknown. Interactions between TM domains have been shown to be important for the folding and assembly of many membrane proteins [ 19, 20]. The structural mechanism has been characterized in greatest depth for the TM domain of glycophorin A (GpA), a dimeric surface protein of erythrocytes. The TM domains of GpA form a highly stable dimer based on close interactions of the two TM helices over an extended segment. Mutagenesis experiments and the nuclear magnetic resonance structure of the GpA TM dimer demonstrated an extended motif within a 13-amino acid segment; seven residues within this segment (LIxxGVxxGVxxT) were shown to make important contributions to dimer formation [ 21– 23]. The key feature of this motif are two glycine residues which permit close interactions between the two TM domains, with the neighboring valine residues of the GVxxGV motif packing into the space available at the glycine positions of the other TM helix. The proximity of the two TM helices also permits formation of rather unusual hydrogen bonds between the backbones of the two TM peptides, specifically between Cα hydrogens from one backbone and carbonyl oxygens of the other backbone [ 24]. The interaction between the two GpA TM peptides is thus stabilized by a large interface with extensive van der Waals interactions as well as hydrogen bonds.

A statistical analysis of TM sequence patterns [ 25] demonstrated that the glycine-based motif (GxxxG) occurs frequently in membrane proteins and that aliphatic amino acids (V, I) are commonly observed at neighboring positions, as first described for GpA. The importance of this motif was further underscored by the analysis of membrane protein crystal structures. A serine-based sequence motif (SxxSSxxT, and variations thereof) has also been described, and again all residues within this motif were found to be critical for TM helix association, reflecting the requirement for a cooperative network of hydrogen bonds [ 26].

Based on this conceptual framework, we investigated whether the assembly of activating immune receptors also involves a structural motif that extends beyond the interaction of the basic TM residue with the pair of acidic residues on the interacting dimer. Surprisingly, large changes within the TM domain did not affect the efficiency of assembly, and even replacement of the entire TM domain with a polyleucine sequence that preserved only the properly positioned basic residue could be tolerated. In contrast to this striking permissiveness along the entire length of the TM helix, a high degree of specificity was observed for the critical basic TM residue, because even exchange of lysine by arginine (KIR receptor) or of arginine by lysine (FcαRI receptor) greatly reduced the efficiency of assembly. Proper assembly therefore appears highly sensitive to the chemical nature of the polar headgroups. Steric hindrance between incompatible extra-membranous domains was identified as another important determinant of assembly specificity.

## Results

### The TM Domains of Receptors That Assemble with DAP12 Are Highly Diverse in Sequence

**Figure 1:**
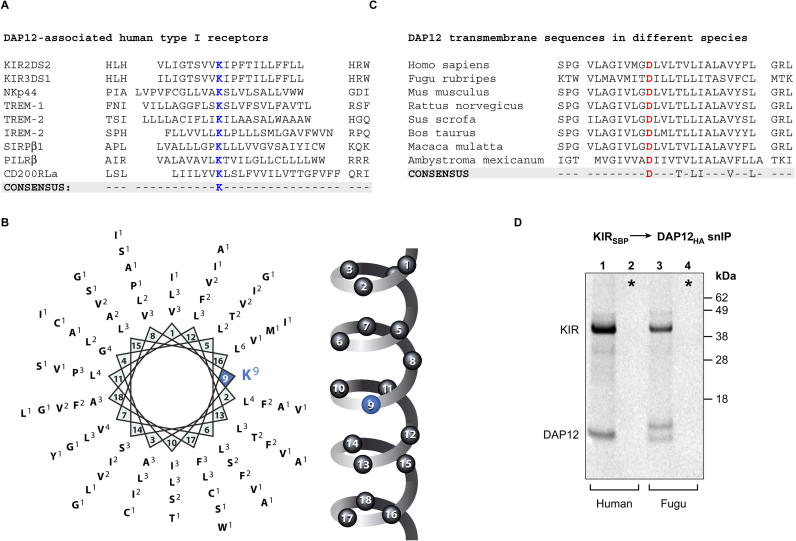
Structural Diversity of TM Domains among Receptors That Assemble with the DAP12 Signaling Dimer (A) Alignment of TM domains and flanking sequences from DAP12-associated human receptors centered on the basic TM residue that is important for receptor assembly with DAP12. (B) Helical wheel representation of the TM residues of DAP12-associated receptors. The frequency of a given amino acid at a particular position is indicated in superscript. (C) Alignment of the DAP12 TM domains and flanking sequences of diverse species from mammals to fish. (D) Assembly of the human KIR receptor with human and puffer fish *(F. rubripes)* DAP12. In vitro assembly reactions were performed as described in Materials and Methods, and were analyzed by snIP targeting SBP-tagged KIR and HA-tagged DAP12. Radiolabeled proteins were separated by SDS-PAGE under reducing conditions, transferred to polyvinylidene fluoride membranes, and exposed to a phosphor imager plate. In control reactions (denoted by asterisks), KIR and DAP12 proteins were translated separately and combined prior to membrane solubilization. The image shown is representative of three experiments.

We previously demonstrated that the TM domain of the human KIR receptor (KIR2DS2) is sufficient for assembly with DAP12 and that the interaction requires the basic TM lysine of KIR and both TM aspartic acid residues of the DAP12 dimer [ 9]. Because the assembly of many other membrane proteins requires specific interactions along extended segments of the TM helices, we examined the TM domains of human receptors that assemble with DAP12 for the presence of an extended motif. The alignment was centered on the basic TM residue in order to determine whether sequence preferences could be identified on the face of the TM helix that interacts with DAP12. The predicted TM domains were 20–23 amino acids in length, and the basic residue was located at position 7–12. The alignment demonstrated that the DAP12-interacting TM domains were highly diverse in sequence ( Figure 1A), and a helical wheel projection ( Figure 1B) failed to identify a higher degree of sequence similarity on the face of the TM helix surrounding the critical basic TM residue compared to the lipid-exposed surface. At a number of positions, the structural diversity was striking because small or large side chains were present, some of which were polar. This analysis revealed that the only common feature beyond the presence of a basic TM residue may be a sufficiently hydrophobic sequence context and the absence of steric hindrance.

Alignment of the TM domain of DAP12 from a variety of different species ranging from Homo sapiens to Fugu rubripes (puffer fish) also demonstrated a substantial level of sequence divergence ( Figure 1C). We therefore examined whether the human KIR receptor (KIR2DS2) could assemble with *Fugu* DAP12 in which only nine of 23 TM residues were conserved to human DAP12. Assembly was examined using an in vitro translation system with endoplasmic reticulum (ER) microsomes, which has previously been shown to reflect faithfully the assembly of complex receptor structures identified in cells using metabolic labeling techniques [ 7, 27]. KIR–DAP12 complexes were isolated from digitonin-solubilized microsomes by two-step sequential non-denaturing immunoprecipitation (snIP) utilizing epitope tags attached to the C-terminus of KIR and DAP12. The streptavidin-binding peptide (SBP) epitope tag attached to KIR permitted elution following the first immunoprecipitation (IP) under non-denaturing conditions by competition with biotin and isolation of intact complexes bearing the DAP12-associated hemagglutinin (HA) tag in the second IP step [ 7, 9]. These experiments demonstrated that the human KIR receptor could assemble with either human or *Fugu* DAP12 ( Figure 1D), suggesting that substantial changes in the TM domains are permitted as long as the required basic/acidic TM residues are present. Similar findings were made with the NKG2C/CD94 receptor that also assembles with DAP12 (unpublished data).

### A Properly Placed Lysine within a Polyvaline or Polyleucine TM Sequence Is Sufficient for Assembly with DAP12

**Figure 2:**
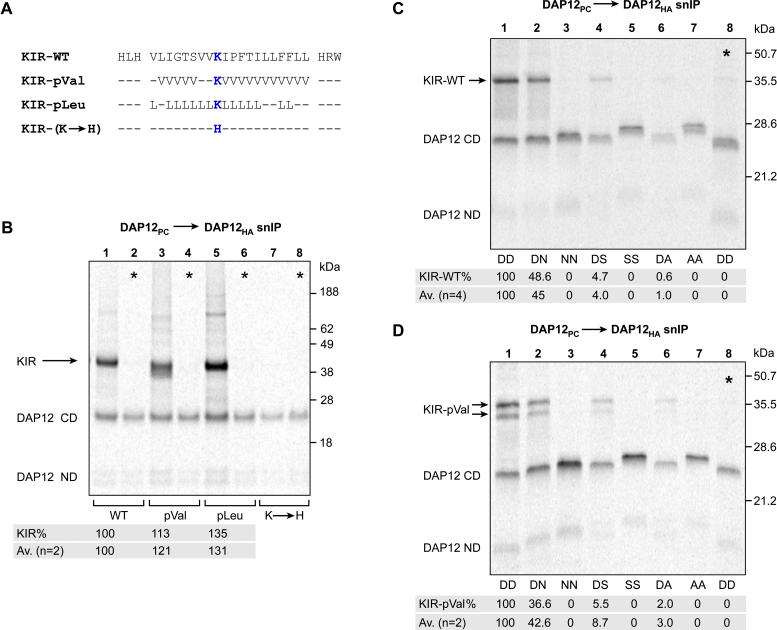
A Properly Placed Lysine within Polyvaline/Polyleucine TM Sequences Is Sufficient for Assembly with DAP12 (A) TM sequences of the KIR mutants utilized in these experiments. The KIR TM domain was replaced with a polyvaline or polyleucine sequence retaining only the native lysine at position 9. In the KIR-(K→H) mutant, the lysine in the TM domain of KIR was substituted by histidine. (B) Assembly of the KIR mutants utilized in these experiments with DAP12. Complexes were isolated by two-step snIP targeting the PC and HA tags attached to the two DAP12 chains. Quantification of DAP12-associated KIR is shown for the average of two separate experiments and adjusted for the total amount of KIR proteins prior to IP. WT KIR was set to 100%. (C and D) Substantial changes in the KIR TM domain do not alter the interaction between the basic TM residue of KIR and the aspartic acid pair of DAP12. Assembly of KIR-WT (C) or KIR-pVal (D) with DAP12 was examined for DAP12 dimers with the WT aspartic acid pair (DD) or mutants in which one or both aspartic acids were substituted by asparagine (N), serine (S), or alanine (A). Each specific DAP12 dimer was isolated by two-step snIP (PC and HA) as in (B). For both KIR-WT and KIR-pVal, no assembly was observed when both DAP12 aspartic acids were substituted (NN, SS, and AA combinations, lanes 3, 5, and 7, respectively). For both KIR forms, a similar pattern was also observed with the set of mutants in which one aspartic acid was substituted (DN, DS, and DA combinations, lanes 2, 4, and 6, respectively). SDS-PAGE was performed under nonreducing conditions. The disulfide-linked DAP12 dimer is labeled as DAP12 CD. A small amount of non-covalent DAP12 dimer (DAP12 ND) was also isolated with this two-step IP method that targeted both DAP12 chains (the two chains dissociate during SDS-PAGE owing to the absence of the interchain disulfide bond). Mixing controls (denoted by asterisk) in lane 8 (C and D) were performed with WT KIR and DAP12. Quantification of DAP12-associated KIR proteins is an average of four (C) and two experiments (D), respectively.

These findings raised the question of whether a basic residue within a hydrophobic sequence context is sufficient for assembly of KIR with DAP12. We therefore replaced the KIR TM segment with a polyvaline or polyleucine sequence into which a single lysine was placed at position 9 ( Figure 2A); these constructs differed from wild-type (WT) only in the TM domain. Isoleucine, leucine, and valine are energetically most favorable in the hydrophobic interior of the plasma membrane [ 28], with valine being less hydrophobic than leucine or isoleucine because of its shorter side chain. Assembly of these KIR mutants was examined by two-step snIP utilizing the protein C (PC) and HA tags attached to the C-terminus of DAP12 chains. Both KIR-pVal and KIR-pLeu assembled with the DAP12 dimer at a similar or higher level compared to WT KIR. In contrast, a point mutation of the KIR TM lysine to histidine completely abolished complex formation ( Figure 2B). Assembly occurred co-translationally because no complex was identified in mixing controls (indicated with asterisk) (lanes 2, 4, 6, and 8, respectively, in Figure 2B) in which DAP12 and KIR chains that had been translated separately were mixed prior to solubilization and IP. These results demonstrated that a high degree of sequence specificity was confined to the KIR TM lysine.

Previous experiments had demonstrated that assembly of KIR involved both TM aspartic acids of the DAP12 dimer [ 9]. Conservative substitution of a single aspartic acid (D) by asparagine (DN combination) impaired assembly, while less-conservative changes of one aspartic acid to serine or alanine (DS and DA combinations) reduced it to very low levels ( Figure 2C). In order to ascertain whether the same interaction between the TM lysine and the aspartic acid pair was formed by the KIR-pVal mutant, we compared assembly of KIR-WT and KIR-pVal with a series of DAP12 mutants in which one or both aspartic acids were mutated to asparagine, serine, or alanine. Assembly products of defined composition were isolated by two-step snIP targeting PC and HA tags attached to the different DAP12 chains, and the amount of co-precipitated KIR was quantitated. The results obtained for the KIR-pVal mutant ( Figure 2D) were strikingly similar to KIR-WT ( Figure 2C), proving that substantial changes in the surrounding TM sequence did not alter the interaction of the KIR lysine with the DAP12 aspartic acid pair.

**Figure 3:**
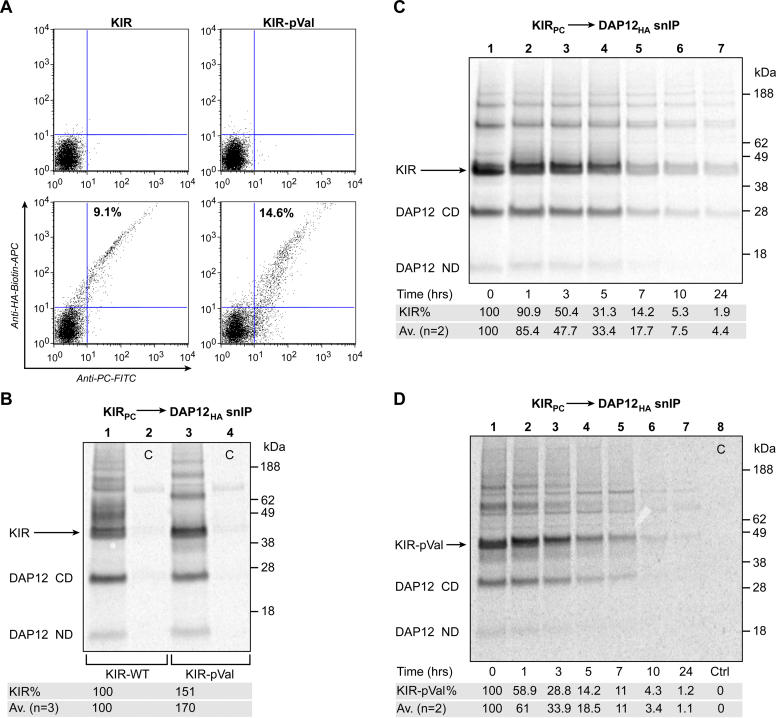
Surface Expression and Half-Life of the Complex between DAP12 and the KIR-pVal Mutant (A) Jurkat cells were transiently transfected with KIR and DAP12 constructs that encoded N-terminal epitope tags. Surface expression of KIR–DAP12 complexes was visualized by FACS utilizing anti-PC mAb plus a fluorescein isothiocyanate–labeled secondary Ab (KIR) as well as a biotinylated anti-HA mAb plus allophycocyanin-labeled streptavidin (DAP12). Surface expression was compared between KIR-WT (left panels) and KIR-pVal (right panels) transfectants stained with isotype control antibodies (upper panels) and HA and PC tag antibodies (lower panels). A larger percentage of cells transfected with KIR-pVal and DAP12 (14.6%) were double-labeled compared to cells transfected with KIR-WT and DAP12 (9.1%). The images shown are representative of four experiments. (B–D) Assembly of KIR-pVal with DAP12 in cells. COS cells transiently transfected with KIR and DAP12 constructs were metabolically labeled and complexes were isolated by two-step snIP targeting KIR-PC and DAP12-HA. (B) Cells were labeled for 30 min and analyzed by PC to HA snIP (lanes 1 and 3) after 3-h chase. In control lanes, the PC mAb was replaced with an isotype control (lanes 2 and 4). (C and D) For comparison of the half-life of complexes formed by KIR-WT or KIR-pVal, cells were again labeled for 30 min, then washed and cultured for 0–24 h. KIR–DAP12 complexes were quantitated following two-step snIP and SDS-PAGE under nonreducing conditions. Quantification is presented for the gels shown as well as the average of two independent experiments.

It was important to demonstrate the relevance of the findings made with the in vitro translation system in cells. We therefore performed metabolic labeling experiments to examine assembly of KIR-pVal with DAP12 in cells as well as fluorescence-activated cell sorter (FACS) analysis to assess expression of the complex at the cell surface ( Figure 3). For that purpose, we transfected Jurkat cells with constructs in which epitope tags were placed at the N-terminus of the extracellular domains of KIR (PC tag) and DAP12 (HA tag). FACS analysis of transiently transfected cells demonstrated surface expression of DAP12 and both KIR forms, with similar expression levels for KIR-pVal and KIR-WT transfectants ( Figure 3A, lower panels) (9.1% and 14.6% of cells transfected with DAP12 and KIR-WT or KIR-pVal, respectively, expressed KIR and DAP12 at the cell surface). The specificity of staining was demonstrated by replacement of epitope tag antibodies with isotype controls ( Figure 3A, upper panels). The diagonal staining pattern in the FACS plots reflects the requirement for co-expression of KIR and DAP12 for efficient transport of these proteins out of the ER [ 13]. Cells transfected with single constructs demonstrated only low levels of the respective proteins at the cell surface (unpublished data).

In order to directly demonstrate formation of KIR–DAP12 complexes, we metabolically labeled transfectants and isolated complexes by two-step snIP utilizing the PC and HA tags attached to KIR and DAP12, respectively ( Figure 3B). These experiments were performed using COS cells because high protein levels can be obtained following transient transfection. KIR–DAP12 complexes were isolated from both KIR-WT and KIR-pVal transfectants, and the specificity of the IP procedure was documented by replacement of the PC tag antibody used in the first step with an isotype control ( Figure 3B, lanes 2 and 4). This IP method was then used to compare the half-life of complexes formed by KIR-WT and KIR-pVal. Transfectants were metabolically labeled for 30 min, washed, and analyzed by two-step IP following a 0 to 24-h chase. The calculated half-lives for KIR-WT and KIR-pVal were 3.06 and 1.78 h, respectively. Three different approaches (in vitro translation system, metabolic labeling of transfectants, and FACS analysis of transfectants) thus demonstrated specific assembly of the KIR-pVal protein with DAP12. Large changes in the TM domain thus do not prevent assembly or transport of the KIR receptor to the cell surface.

### Large Changes Can Also Be Made in the TM Domain of the Unrelated NKG2D Receptor

These results raised the question of whether insensitivity to large changes within the TM domain was restricted to the KIR receptor, or whether it was a more general property of receptors that assemble with their signaling modules through interaction of basic and acidic TM residues. We examined the NKG2D receptor utilized by NK cells for detection of stress-regulated surface proteins because it belongs to a different protein family than KIR (C-type lectin rather than immunoglobulin), associates with a different signaling dimer (DAP10 rather than DAP12), and has a different membrane topology (type II rather than type I) [ 1, 12]. Previous experiments had demonstrated that the NKG2D and DAP10 TM domains were sufficient for assembly [ 8].

**Figure 4:**
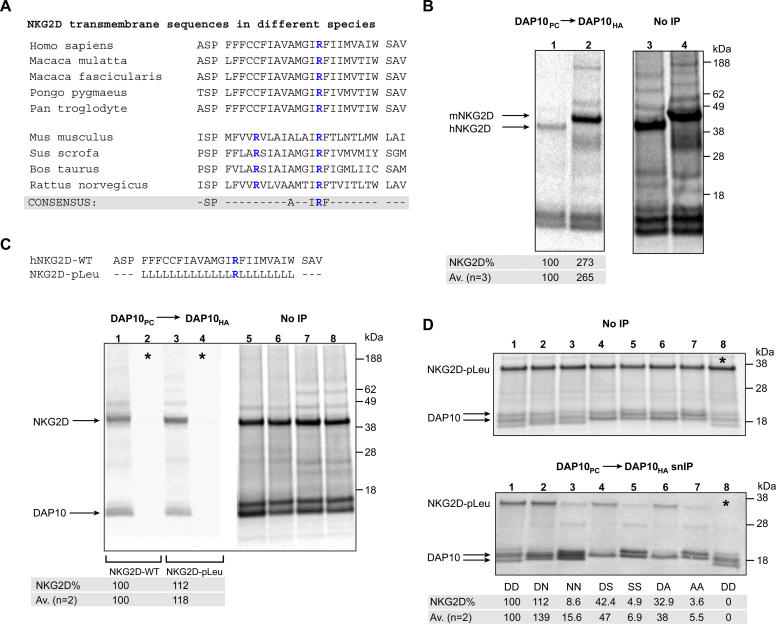
Structural Requirements for Assembly of DAP10 with NKG2D, a Type II TM Receptor (A) Sequences of TM domains and flanking segments for NKG2D in different species. (B) Human DAP10 assembly with human or mouse NKG2D (lanes 1 and 2, respectively). Complexes were isolated by two-step snIP utilizing PC and HA tags attached to DAP10, and associated NKG2D was quantitated. (C) Efficient assembly of DAP10 with NKG2D-pLeu in which the native TM domain was substituted with a polyleucine sequence containing a properly positioned arginine. Assembly of DAP10 with NKG2D proteins was examined by two-step snIP as in (B). The images shown are representative of two experiments. (D) Modification of the TM domain in NKG2D-pLeu does not alter the interaction between the TM arginine of NKG2D and the aspartic acid pair of DAP10. Images were analyzed as described in Figure 2C for KIR-DAP12. SDS-PAGE was performed under reducing conditions.

Alignment of the NKG2D TM domains from different species demonstrated that they fell into two groups which had only four TM residues in common ( Figure 4A). We selected the murine NKG2D sequence as a representative of the second group and examined whether this protein could assemble with human DAP10. DAP10 dimer-associated proteins were isolated by two-step snIP utilizing the PC and HA tags attached to DAP10 chains. Surprisingly, murine NKG2D assembled with human DAP10 more efficiently (˜2.7-fold) than human NKG2D, indicating that large changes in the NKG2D TM domain were tolerated ( Figure 4B). Because none of the NKG2D TM residues are leucines, replacement of the TM domain by a polyleucine sequence with a single arginine represented a drastic change of the TM domain. Nevertheless, this NKG2D-pLeu protein assembled with DAP10 at an efficiency that was remarkably similar to WT NKG2D ( Figure 4C). Furthermore, the TM arginine formed the same interaction with the aspartic pair of DAP10, as shown by analysis of a set of DAP10 mutants in which one or both aspartic acids were substituted with polar or nonpolar residues ( Figure 4D). This experiment was performed as described in Figure 2D for the KIR–DAP12 complex, and yielded a pattern for the NKG2D-pLeu protein similar to WT NKG2D [ 8].

We also replaced the TM domain of NKG2C with a polyleucine sequence and a properly positioned lysine, and found that this protein assembled with DAP12 at a similar level as WT NKG2C, indicating that formation of the three-helix interface was again not affected by large changes of the TM sequence. However, formation of the heterodimer between NKG2C and CD94 was reduced by replacement of the TM sequence (unpublished data). The FcαRI assembles with the Fcγ signaling module, and an FcαRI mutant in which the TM domain was replaced with a polyleucine sequence with an arginine at position 3 assembled with Fcγ, albeit at a reduced level (˜11% relative to WT FcαRI; unpublished data). For three out of four receptors studied, large changes of the TM sequence therefore did not reduce assembly of the ligand-binding subunit carrying the basic TM residue with the relevant signaling module.

### DAP12 and Fcγ Signaling Dimers Have a Strong Preference for a Particular Basic TM Side Chain

**Figure 5:**
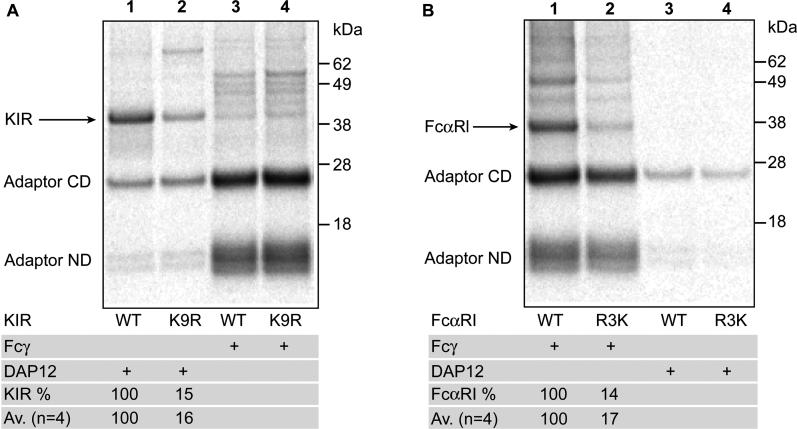
Mechanisms of Specificity I: Preference for a Particular Basic TM Residue The KIR receptor has a lysine close to the center of the TM domain (position 9), while the FcαRI receptor carries an arginine in the N-terminal segment of the TM domain (position 3). (A) Substitution of the KIR TM lysine by arginine (K9R) substantially reduced assembly with DAP12 (lanes 1 and 2). No assembly was observed with the Fcγ signaling dimer (lanes 3 and 4). (B) Substitution of the FcαRI receptor TM arginine by lysine (R3K) substantially reduced assembly with Fcγ (lanes 1 and 2). No assembly was observed with the DAP12 signaling dimer (lanes 3 and 4). SDS-PAGE was performed under nonreducing conditions. DAP12-associated KIR (A) or Fcγ-associated FcαRI (B) quantitations were normalized to total translation levels of these proteins. The images shown are representative of four experiments.

The results presented above demonstrate that the surrounding TM sequence contributes relatively little to the specificity of assembly and raises the question of how formation of inappropriate complexes between receptors and signaling dimers is prevented. DAP12 and Fcγ are expressed by many different cell types of hematopoietic origin and are signaling components of a variety of different receptors, as described in the Introduction. We examined whether these signaling modules have a preference for a particular basic TM residue, because receptors that assemble with Fcγ have an arginine in their TM domain, while all type I membrane proteins known to associate with human DAP12 have a TM lysine. Mouse and rat DAP12 also assemble with the type II membrane protein Ly49, which has a TM arginine [ 29]. Substitution of the KIR TM lysine by arginine (K9R mutant) reduced assembly to ˜15% compared to KIR-WT ( Figure 5A). Remarkably, a similar reduction in assembly efficiency with Fcγ was observed when the converse change from arginine to lysine was made in the TM domain of FcαRI ( Figure 5B). However, substitution of the TM arginine of NKG2D by lysine did not reduce the efficiency of assembly with DAP10 (unpublished data).

Neither KIR nor FcαRI associated with the inappropriate signaling dimer ( Figures 5A and B, lanes 3 and 4). For FcαRI, this finding is explained by the mismatch in the location of the ionizable TM residues—position 3 of the predicted TM domain for the arginine of FcαRI relative to position 9 for the aspartic acid of DAP12. For KIR, the TM aspartic acid pair of Fcγ (position 6) is located within a closer distance to the TM lysine (position 9), but an interaction could nevertheless not be detected. The high degree of specificity for lysine versus arginine required for interaction of these type I membrane proteins with the DAP12 and Fcγ signaling modules, respectively, is in striking contrast to the flexibility of sequence requirements at all other TM positions.

### Specificity of Assembly Can Result from Steric Hindrance between Incompatible Extracellular Domains

**Figure 6:**
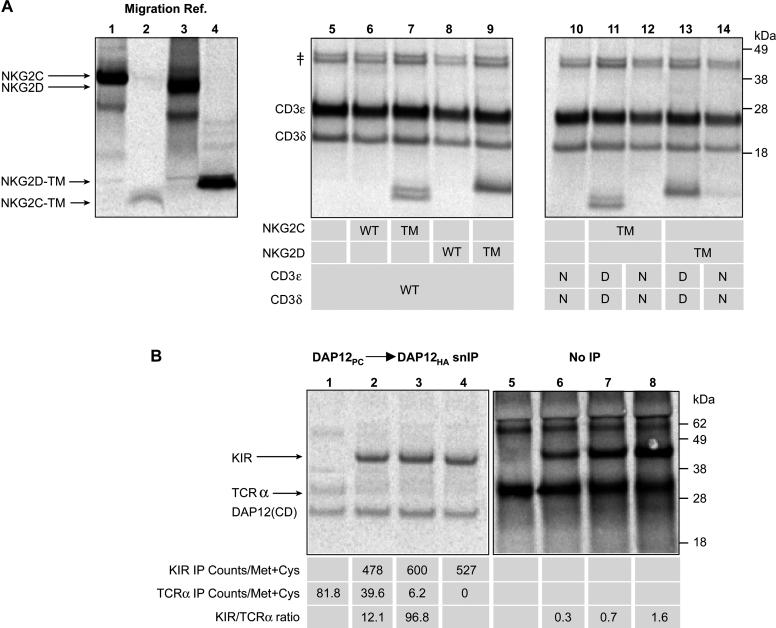
Mechanisms of Specificity II: Steric Hindrance and Competition for Assembly Partners (A) Steric hindrance between incompatible extracellular domains. Assembly of full-length NKG2C and NKG2D (WT) and the TM peptides of these two receptors (TM) with CD3δ was examined using an anti-CD3 mAb (UCH-T1) for IP analysis. The TM peptides, but not the full-length proteins, assembled with CD3δ (lanes 5–9). The interaction required the TM aspartic acid pair of CD3δ based on the analysis of mutants in which the aspartic acids (D) were substituted with asparagine (N) (lanes 10–14). The migration of the individual proteins is demonstrated in lanes 1–4 as a reference. A nonspecific dimer of CD3 is marked with a cross. (B) The KIR receptor out-competes an irrelevant receptor for DAP12-binding. All in vitro translation experiments were set up with 100 ng of DAP12 RNA and 150 ng of TCRα RNA, while 0, 50, 100, or 150 ng of KIR RNA was added to reactions 1–4. In the reaction without KIR (lane 1), a small amount of TCRα associated with the DAP12 dimer. In the presence of increasing concentrations of KIR (lanes 2–4), TCRα binding to DAP12 was lost. Complex formation was analyzed by two-step snIP targeting the PC and HA tags attached to DAP12. KIR and TCRα were quantitated following IP (lanes 1–4) as well as from aliquots of reactions not subjected to IP (lanes 5–8). IP counts were divided by the number of methionine and cysteine residues to reflect the molar ratio of KIR and TCRα chains, which is shown both before and following IP. SDS-PAGE was performed under nonreducing conditions. The images shown are representative of three experiments.

Previous experiments had shown that the TM peptides of KIR and NKG2D were necessary and sufficient for assembly with DAP12 and DAP10, respectively [ 8, 9]. Given the small contributions of neighboring TM residues to specificity of assembly, we examined whether isolated TM peptides could form complexes with inappropriate signaling modules. Specificity of assembly is particularly relevant for the TCR–CD3 complex because T cell activation is triggered by ligation of very few receptors [ 30]. The NKG2D protein is expressed in subsets of T cells, but provides only a co-stimulatory signal that amplifies activation through the TCR [ 31, 32]. Inappropriate assembly of NKG2D with TCR signaling modules would thus seriously affect T cell function. We therefore compared the ability of full-length NKG2D and NKG2D TM peptides to assemble with CD3δɛ. The NKG2D TM construct encoded an initiation methionine, the TM domain, and several N- and C-terminal flanking residues, as well as a C-terminal epitope tag. This TM peptide efficiently assembled with CD3δɛ, but no assembly was observed for full-length NKG2D ( Figure 6A, lanes 8 and 9). The interaction of the NKG2D TM peptide required the TM aspartic acid pair of CD3δɛ because substitution of both residues by asparagine interfered with complex formation ( Figure 6A, lanes 13 and 14). Very similar findings were made with full-length NKG2C versus a NKG2C TM peptide ( Figure 6A, lanes 6, 7, 11, and 12). The extra-membranous domains thus prevented formation of inappropriate complexes through steric hindrance.

### Competition for Assembly Partners Can Contribute to Specificity of Assembly

The DAP12 signaling dimer is expressed in subsets of T cells [ 33], and its aspartic acid pair is located at the same position as the corresponding pair of acidic residues in the TCR-interacting CD3δɛ and CD3γɛ dimers. In assembly reactions in which KIR was not present, small quantities of TCRα were found to associate with DAP12. However, KIR out-competed TCRα for assembly with DAP12 even when present at lower levels than TCRα ( Figure 6B). Even at a ratio of total KIR to TCRα protein of 0.7, virtually all DAP12 molecules associated with KIR rather than TCRα ( Figure 6B, lane 3). Competition for signaling modules can therefore also contribute to specificity of assembly.

## Discussion

**Figure 7:**
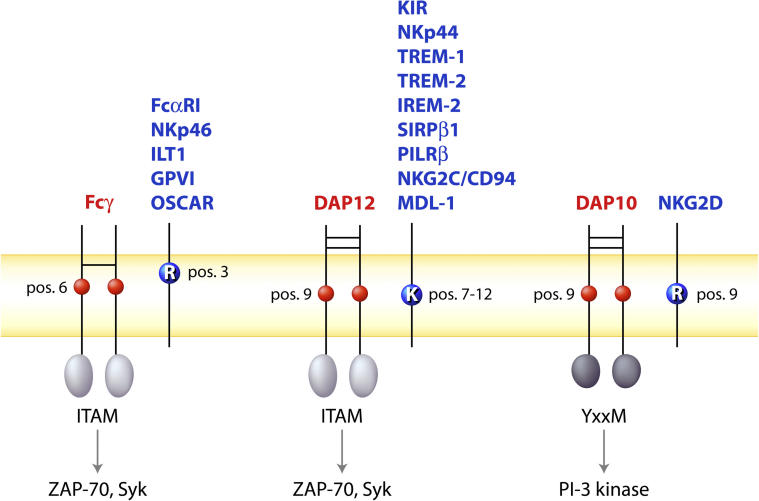
Mechanisms for Selective Assembly of a Diverse Group of Receptors with their Signaling Modules The Fcγ and DAP12 signaling modules are expressed by a variety of different cell types of hematopoietic origin and serve as signaling subunits for many different receptors. Fcγ and DAP12 exhibit a preference for a particular basic side chain, Fcγ for arginine (R) and DAP12 for lysine (K). The location of the ionizable TM residues also contributes to specificity of assembly, an aspect that is important because Fcγ and DAP12 are co-expressed by a number of different cell types. The DAP10 signaling dimer assembles only with NKG2D and initiates signaling through interaction with the p85 subunit of PI-3 kinase, while the other two adaptors signal through ZAP-70 or Syk tyrosine kinases.

A highly focused interaction between three ionizable TM residues thus determines the assembly of a diverse group of receptors with their dimeric signaling modules. Particularly striking is the high sensitivity of the assembly process to the precise chemical nature of the basic head group, as shown by substantial reduction of assembly efficiency following lysine to arginine (KIR–DAP12 interaction) or arginine to lysine (FcαRI–Fcγ interaction) substitution. These results explain why all receptors with a basic TM residue that associate with Fcγ carry a TM arginine, while all receptors with a type I membrane topology that assemble with human DAP12 have a properly positioned TM lysine ( Figure 7). Mouse and rat DAP12 assemble with Ly49 receptors with a TM arginine (type II membrane proteins) [ 29], and it is possible that an arginine is more favorable within a TM helix with a type II rather than type I topology. The position of the basic TM residue also contributes to specificity of assembly: in all receptors that assemble with Fcγ, the arginine is located in the N-terminal segment of the TM domain, while the TM lysine residue of DAP12-interacting proteins is positioned closer to the center of the TM domains, matching the location of the aspartic acid pair in the respective signaling dimer [ 34] ( Figure 7). The majority—but not all—activating receptors expressed by distinct cell types of hematopoietic origin associate with their signaling modules utilizing a basic TM residue. Exceptions include the FcRI and CD16 receptors that associate with Fcγ, but lack a basic TM residue [ 18, 35]. The structural interactions responsible for assembly of these complexes have not been defined in detail.

The complete exchangeability of the TM sequence surrounding the basic residue is in striking contrast to the specificity for a particular basic side chain. The only requirements for the surrounding TM sequence appear to be a lack of steric hindrance and a sufficient degree of hydrophobicity. The degree of hydrophobicity is particularly relevant for type II membrane proteins such as NKG2D, and a NKG2D construct with a polyleucine TM sequence and a TM arginine inserted into the lipid bilayer with the correct type II topology ( Figure 4C), while the less hydrophobic polyvaline variant adopted an inappropriate type I orientation (unpublished data). Even though the neighboring TM sequence plays only a secondary role in assembly, it is possible that it contributes to the function of some of these receptor complexes at the cell surface.

These unique polar interactions in the membrane appear to provide sufficient stability to a variety of different receptors without constraining ligand interactions by extracellular domains or assembly of large signaling complexes on cytoplasmic domains. Polar residues make much larger energetic contributions to protein–protein interactions in the hydrophobic interior of the lipid bilayer than in an aqueous environment because of the lower dielectric environment in the membrane. In contrast, sequestration of a hydrophobic residue at a protein–protein interface makes a smaller energetic contribution in a hydrophobic than in an aqueous environment [ 19, 20, 36]. It is thus conceivable that the three ionizable residues make the dominant energetic contribution to the assembly of the activating immune receptors discussed here. This notion is consistent with the finding that introduction of a single residue with a carboxyl or carboxamide side chain (aspartic acid, glutamic acid, asparagine, or glutamine) into model TM helices can result in formation of sodium dodecyl sulfate (SDS)–stable homodimers or homotrimers [ 37, 38]. In addition, electrostatic attraction occurs over much larger distances within membranes compared to an aqueous environment—an aspect that may facilitate efficient interaction between receptors and signaling modules [ 36].

Given the apparent strength of these polar interactions, it is important to consider the mechanisms ensuring specificity of assembly. Specificity is critical because these receptors control the activation state of all of the different cell types in the immune system, and inappropriate activation of such cells could result in chronic inflammation or autoimmunity. Experiments with the NKG2D TM peptide demonstrated the potential for inappropriate interactions with the CD3δɛ and CD3γɛ dimers which associate in vivo only with the TCR. However, no assembly of either CD3 dimer with full-length NKG2D protein was observed, indicating that the extra-membranous domains of NKG2D prevented such undesirable interactions through steric hindrance. In previous experiments, we and others had found that the extracellular domains of the CD3δɛ and CD3γɛ dimers contribute to the specificity TCR–CD3 assembly: replacement of the CD3γ TM domain with the CD3δ TM domain was tolerated (both of which have an acidic residue at the same position), while the corresponding replacement of the CD3γ extracellular domain prevented formation of the complex [ 7, 39]. Other mechanisms of specificity are cell type–specific expression of particular receptors, such as T cell–specific expression of TCRα and β or TCRγ and δ chains, competition among receptors for the appropriate signaling dimers (as shown in Figure 6), and steric hindrance between TM domains (as reported for NKG2D [ 40]).

Both DAP12 and Fcγ serve as signaling modules for a variety of different receptors, and the highly focal nature of the interaction explains why many receptors with diverse TM sequences can assemble with the same signaling dimer. Furthermore, it accounts for the fact that two large protein families independently adopted this assembly mechanism. The KIR and NKG2C/CD94 receptors belong to the immunoglobulin and C-type lectin protein families and adopt a type I and type II membrane topology [ 29], respectively, proving that their DAP12-interacting TM domains did not originate from a common ancestor gene [ 9]. Both receptors have inhibitory counterparts that lack the TM lysine and that are evolutionarily more ancient [ 41]. The ability to assemble with DAP12 is thus the result of independent mutations in TM domains that introduced a lysine at a position suitable for binding to DAP12.

It is also apparent that the TM arginine located in the N-terminal part of the TM domain of Fcγ-interacting receptors arose independently from the TM lysine of DAP12-interacting receptors located closer to the center of the TM helix. It is therefore likely that this assembly mechanism evolved separately in at least three instances, favored by the focal nature of the changes required for association with existing signaling modules. In this context, it is important to note that the DAP10, DAP12, Fcγ, and ζ signaling modules are evolutionarily more ancient than the KIR, NKG2C/CD94, or NKG2D receptors [ 41] because they are present in several orders of mammals, as well as in amphibians and bony fishes, while the NK receptors described above have been identified only in mammals. The widespread utilization of this assembly mechanism may thus be the result of point mutations in TM domains that yielded receptors with new functionality. Studies on the murine Ly49 genes strongly suggest pathogen-driven evolution as the mechanism [ 42].

## Materials and Methods

### cDNA constructs for in vitro translation experiments

The protein sequences of all constructs are shown in Dataset S1. All constructs for in vitro translation experiments were cloned into a modified pSP64 vector (provided by M. Kozak, UMDNJ-RW Johnson Medical School, Piscataway, New Jersey, United States), and native signal peptides of type I membrane proteins were replaced by the murine H-2K ^b^ signal sequence. Epitope tags were added as in-frame fusions, usually with a three-amino acid flexible linker [ 7]. The murine NKG2D construct was generated by RT-PCR using mRNA from splenocytes and cloned into the pSP64 vector with a C-terminal SBP tag. The cDNA construct for *Fugu* DAP12 was assembled from synthetic oligonucleotides with C-terminal PC or HA tags. The EC domains of human DAP12, *Fugu* DAP12, and DAP10 carry two cysteines, and we previously showed that only one of these was required to achieve WT levels of disulfide-linked dimers [ 9]. The first of these two cysteines in the EC domain was therefore mutated to serine to prevent formation of disulfide-linked multimers; in addition, the cysteine in the C-terminal part of the *Fugu* DAP12 TM domain was mutated to serine. Mutations were introduced into previously established KIR, NKG2D, and FcαRI constructs [ 9] by overlapping PCR. All other constructs were as previously described.

### In vitro transcription and translation

In vitro transcription was performed from linearized cDNA constructs using the RiboMax T7 large-scale RNA production kit and m ^7^G cap analog (Promega; Madison, Wisconsin, United States). Each 25-μl translation reaction contained 17.5 μl nuclease-treated rabbit reticulocyte lysate (Promega), 0.5 μl amino acid mixture minus methionine/cysteine (Promega), 0.5 μl SUPERase-In RNase inhibitor (Ambion, Austin, Texas, United States), 1–2 μl [ ^35^S]–labeled methionine/cysteine (Pro-Mix, Amersham Biosciences, Little Chalfont, United Kingdom), RNA (200 ng for receptors and 100–200 ng for adaptor chains, unless otherwise noted), and 2.0 μl ER microsomes from a murine hybridoma (IVD12) which were isolated as previously described [ 7]. All in vitro translation and assembly reactions were performed at 30 °C. An initial translation period of 30 min under reducing conditions was followed by a 2-h assembly period (4 h for NKG2D and DAP10) after addition of oxidized glutathione to 4 mM. Reaction volumes were 25–75 μl as required for optimal signal with multi-step snIP procedures.

### IP, electrophoretic analysis, and densitometry

The following mAbs directed against epitope tags were used for IP procedures: high-affinity anti-HA (rat mAb 3F10) and calcium-dependent anti-PC (mouse mAb HPC4) from Roche (Indianapolis, Illinois, United States), as well as anti-CD3 (mouse mAb UCH-T1) from Santa Cruz (Santa Cruz, California, United States).

Translation and assembly reactions were stopped with 1 ml ice-cold Tris-buffered saline (TBS)/10 mM iodoacetamide, and microsomes were pelleted (10 min/20,000 *g*/4 °C) and rinsed. Pellets were solubilized in 400 μl IP buffer (TBS plus 0.5% digitonin [Biosynth International, Naperville, Illinois, United States], 10 mM iodoacetamide, 0.1% BSA, 5 μg/ml leupeptin, and 1 mM PMSF; with 1 mM CaCl _2_ when anti-PC mAb was used) for 30 min rotating at 4 °C. Lysates were precleared for 1 h with Tris/BSA-blocked protein G-Sepharose 4 beads (Amersham Biosciences), and primary captures were performed overnight at 4 °C. Primary IP products were washed twice in 0.5 ml washing buffer (TBS plus 0.5% digitonin and 10 mM iodoacetamide, with 1 mM CaCl _2_ for anti-PC mAb-binding). Non-denaturing elution with EDTA (PC tag) or biotin (SBP tag) was performed as described [ 7], and eluted complexes were incubated with subsequent antibodies and protein G-Sepharose 4 beads for 2 h at 4 °C and washed. Final precipitates were digested for 1 h at 37 °C with 500 U endoglycosidase H (New England Biolabs, Beverly, Massachusetts, United States), separated on 12% NuPAGE Bis-Tris gels (Invitrogen, Carlsbad, California, United States), transferred to polyvinylidene fluoride membranes, and exposed to phosphor imager plates. Gels in Figures 1D and 4B–D were run under reducing conditions; all other gels were run under nonreducing conditions. Densitometry was performed using the Wide Line Tool in the ImageQuant software package (Molecular Dynamics, Sunnyvale, California, United States).

### Transfection of KIR and DAP12 constructs into Jurkat cells

KIR, KIR-pVal, and DAP12 constructs were cloned into the pCI-neo expression vector (Promega) with epitope tags placed at the N-terminus of mature proteins (PC tag for KIR and KIR-pVal, and HA tag for DAP12) so that surface expression could be assessed following transfection. KIR plasmids were co-transfected with DAP12 into Jurkat cells by electroporation. Plasmid DNA was isolated by cesium chloride density-gradient centrifugation; 20 μg DNA of each construct and 1 × 10 ^7^ cells were used per electroporation (400-μl volume; 250 V/950 μF in 0.4-cm gap cuvettes). Cells were then transferred to warm DMEM medium supplemented with 10% fetal bovine serum, 2 mM glutamine, 100 IU penicillin, 100 μg/ml streptomycin, and 10 mM HEPES. Following overnight culture, live cells were isolated by Ficoll density-gradient centrifugation. For FACS analysis, cells were labeled with either anti-PC and biotinylated anti-HA tag mAbs or with isotype controls in the presence of 5 mM calcium. Second-step reagents were fluorescein isothiocyanate–labeled anti-mouse IgG1 (PC antibody) and allophycocyanin-labeled streptavidin (HA antibody) (BD Bioscience, Franklin Lakes, New Jersey, United States). Flow-cytometric analysis was done using a FACSCalibur (Becton Dickinson Immunocytometry Systems, San Jose, California, United States).

### Metabolic labeling and IP

COS cells were co-transfected with KIR and DAP12 constructs using the lipofectamine 2000 reagent (Invitrogen) and cultured for 24 h at 37 °C. Cells were then incubated in cysteine- and methionine-deficient media (DMEM supplemented with dialyzed FCS) for 15 min at 37 °C, washed, and pulse-labeled with ^35^S-labeled methionine and cysteine for 30 min at 37 °C (400 μCi ^35^S Pro-Mix [Amersham] in 2 ml for 6 × 10 ^6^ cells). Cells were then washed and cultured for 0–24 h in DMEM medium supplemented with 10% fetal bovine serum, 2 mM glutamine, 100 IU penicillin, 100 μg/ml streptomycin, and 10 mM HEPES. Following chase, cells were solubilized as described above. Complexes were isolated by two-step snIP utilizing the PC tag attached to KIR and the HA tag attached to DAP12, and subjected to SDS-PAGE under nonreducing conditions. Transfer to polyvinylidene fluoride membranes and densitometry were performed as described above.

## Supporting Information

Dataset S1 Protein Sequences of Constructs Used in Assembly Experiments (46 KB DOC)

### Accession Numbers

The GenBank ( http://www.ncbi.nlm.nih.gov/Genbank) accession numbers for the genes and gene products discussed in this paper are CD3δ (AH002612 and AH002678), DAP12 (AF019563), FcαRI (X54150), Fcγ signaling module (M33195), *Fugu* DAP12 (CAAB01002552), human DAP10 (AF285447), human KIR receptor KIR2DS2 (NM_012312), murine NKG2D sequence (AF030313), NKG2C/CD94 receptor (AJ001684 and U30610), and human NKG2D receptor (AF461811).

## Abbreviations

ER: endoplasmic reticulum
FACS: fluorescence-activated cell sorter
GpA: glycophorin A
HA: hemagglutinin
IP: immunoprecipitation
NK: natural killer
PC: protein C
SBP: streptavidin-binding peptide
SDS: sodium dodecyl sulfate
snIP: sequential non-denaturing immunoprecipitation
TBS: Tris-buffered saline
TCR: T cell receptor
TM: transmembrane
WT: wild-type
