# Supplementary material for: The Assembly of Diverse Immune Receptors Is Focused on a Polar Membrane-Embedded Interaction Site
Source: PLoS Biol. 2006 Apr 25;4(5):e142. doi: 10.1371/journal.pbio.0040142 (PMC1440944; doi:10.1371/journal.pbio.0040142)
Supplement: Supplementary file 1 [file pbio.0040142.sd001.doc]

**Protein Sequences:**

**A. constructs for in vitro translation experiments**

KIR2DS2.SBP tag

```
1      MVPCTLLLLL AAALAPTQTR AHEGVHRKPS LLAHPGPLVK SEETVILQCW SDVRFEHFLL
61     HREGKYKDTL HLIGEHHDGV SKANFSIGPM MQDLAGTYRC YGSVTHSPYQ LSAPSDPLDI
121    VITGLYEKPS LSAQPGPTVL AGESVTLSCS SRSSYDMYHL SREGEAHERR FSAGPKVNGT
181    FQADFPLGPA THGGTYRCFG SFRDSPYEWS NSSDPLLVSV TGNPSNSWPS PTEPSSKTGN
241    PRHLHVLIGT SVVKIPFTIL LFFLLHRWCS NKKNAAVMDQ EPAGNRTVNS EDSDEQDHQE
301    VSYAGSDEKT TGWRGSGHVV EGLAGELEQL RARLEHHPQG QREPSSSGGS KLG
```

KIR2DS2-pVal.SBP tag

```
1      MVPCTLLLLL AAALAPTQTR AHEGVHRKPS LLAHPGPLVK SEETVILQCW SDVRFEHFLL
61     HREGKYKDTL HLIGEHHDGV SKANFSIGPM MQDLAGTYRC YGSVTHSPYQ LSAPSDPLDI
121    VITGLYEKPS LSAQPGPTVL AGESVTLSCS SRSSYDMYHL SREGEAHERR FSAGPKVNGT
181    FQADFPLGPA THGGTYRCFG SFRDSPYEWS NSSDPLLVSV TGNPSNSWPS PTEPSSKTGN
241    PRHLHVVVVV VVVKVVVVVV VVVVVHRWCS NKKNAAVMDQ EPAGNRTVNS EDSDEQDHQE
301    VSYAGSDEKT TGWRGSGHVV EGLAGELEQL RARLEHHPQG QREPSSSGGS KLG
```

KIR2DS2-pLeu.SBP tag

```
1      MVPCTLLLLL AAALAPTQTR AHEGVHRKPS LLAHPGPLVK SEETVILQCW SDVRFEHFLL
61     HREGKYKDTL HLIGEHHDGV SKANFSIGPM MQDLAGTYRC YGSVTHSPYQ LSAPSDPLDI
121    VITGLYEKPS LSAQPGPTVL AGESVTLSCS SRSSYDMYHL SREGEAHERR FSAGPKVNGT
181    FQADFPLGPA THGGTYRCFG SFRDSPYEWS NSSDPLLVSV TGNPSNSWPS PTEPSSKTGN
241    PRHLHLLLLL LLLKLLLLLL LLLLLHRWCS NKKNAAVMDQ EPAGNRTVNS EDSDEQDHQE
301    VSYAGSDEKT TGWRGSGHVV EGLAGELEQL RARLEHHPQG QREPSSSGGS KLG
```

NKG2C.SBP tag

```
1      MSKQRGTFSE VSLAQDPKRQ QRKPKGNKSS ISGTEQEIFQ VELNLQNPSL NHQGIDKIYD
61     CQGLLPPPEK LTAEVLGIIC IVLMATVLKT IVLIPFLEQN NSSPNTRTQK ARHCGHCPEE
121    WITYSNSCYY IGKERRTWEE SLLACTSKNS SLLSIDNEEE MKFLASILPS SWIGVFRNSS
181    HHPWVTINGL AFKHKIKDSD NAELNCAVLQ VNRLKSAQCG SSMIYHCKHK LGSDEKTTGW
241    RGSGHVVEGL AGELEQLRAR LEHHPQGQRE PSSSGGSKLG
```

NKG2C-TM.SBP tag

```
1      MGSDEKTTGW RGGHVVEGLA GELEQLRARL EHHPQGQREP SSSGGSKLGG STAEVLGIIC
61     IVLMATVLKT IVLIPFLEQN
```

CD94

```
1      MAVFKTTLWR LISGTLGIIC LSLMATLGIL LKNSFTKLSI EPAFTPGPNI ELQKDSDCCS
61     CQEKWVGYRC NCYFISSEQK TWNESRHLCA SQKSSLLQLQ NTDELDFMSS SQQFYWIGLS
121    YSEEHTAWLW ENGSALSQYL FPSFETFNTK NCIAYNPNGN ALDESCEDKN RYICKQQLI
```

NKG2D.SBP tag

```
1      MGWIRGRRSR HSWEMSEFHN YNLDLKKSDF STRWQKQRCP VVKSKCRENA SPFFFCCFIA
61     VAMGIRFIIM VAIWSAVFLN SLFNQEVQIP LTESYCGPCP KNWICYKNNC YQFFDESKNW
121    YESQASCMSQ NASLLKVYSK EDQDLLKLVK SYHWMGLVHI PTNGSWQWED GSILSPNLLT
181    IIEMQKGDCA LYASSFKGYI ENCSTPNTYI CMQRTVGSDE KTTGWRGGHV VEGLAGELEQ
241    LRARLEHHPQ GQREPSSSGG SKLG
```

NKG2D-pLeu.SBP tag

```
1      MGWIRGRRSR HSWEMSEFHN YNLDLKKSDF STRWQKQRCP VVKSKCRENA SPLLLLLLLL
61     LLLLLRLLLL LLLLSAVFLN SLFNQEVQIP LTESYCGPCP KNWICYKNNC YQFFDESKNW
121    YESQASCMSQ NASLLKVYSK EDQDLLKLVK SYHWMGLVHI PTNGSWQWED GSILSPNLLT
181    IIEMQKGDCA LYASSFKGYI ENCSTPNTYI CMQRTVGSDE KTTGWRGGHV VEGLAGELEQ
241    LRARLEHHPQ GQREPSSSGG SKLG
```

NKG2D-TM.SBP tag

```
1      MENASPFFFC CFIAVAMGIR FIIMVAIWSA VFLGSDEKTT GWRGGHVVEG LAGELEQLRA
61     RLEHHPQGQR EPSSSGGSKL G
```

Mouse NKG2D.SBP tag

```
1      MALIRDRKSH HSEMSKCHNY DLKPAKWDTS QEQQKQRLAL TTSQPGENGI IRGRYPIEKL
61     KISPMFVVRV LAIALAIRFT LNTLMWLAIF KETFQPVLCN KEVPVSSREG YCGPCPNNWI
121    CHRNNCYQFF NEEKTWNQSQ ASCLSQNSSL LKIYSKEEQD FLKLVKSYHW MGLVQIPANG
181    SWQWEDGSSL SYNQLTLVEI PKGSCAVYGS SFKAYTEDCA NLNTYICMKR AVGSDEKTTG
241    WRGGHVVEGL AGELEQLRAR LEHHPQGQRE PSSSGGSKLG
```

FcαRI.SBP tag

```
1      MVPCTLLLLL AAALAPTQTR AQEGDFPMPF ISAKSSPVIP LDGSVKIQCQ AIREAYLTQL
61     MIIKNSTYRE IGRRLKFWNE TDPEFVIDHM DANKAGRYQC QYRIGHYRFR YSDTLELVVT
121    GLYGKPFLSA DRGLVLMPGE NISLTCSSAH IPFDRFSLAK EGELSLPQHQ SGEHPANFSL
181    GPVDLNVSGI YRCYGWYNRS PYLWSFPSNA LELVVTDSIH QDYTTQNLIR MAVAGLVLVA
241    LLAILVENWH SHTALNKEAS ADVAEPSWSQ QMCQPGLTFA RTPSVCKGSD EKTTGWRGGH
301    VVEGLAGELE QLRARLEHHP QGQREPSSSG GSKLG
```

TCRα

```
1      MVPCTLLLLL AAALAPTQTR AQKEVEQNSG PLSVPEGAIA SLNCTYSDRG SQSFFWYRQY
61     SGKSPELIMS IYSNGDKEDG RFTAQLNKAS QYVSLLIRDS QPSDSATYLC AVTTDSWGKL
121    QFGAGTQVVV TPDIQNPDPA VYQLRDSKSS DKSVCLFTDF DSQTNVSQSK DSDVYITDKT
181    VLDMRSMDFK SNSAVAWSNK SDFACANAFN NSIIPEDTFF PSPESSCDVK LVEKSFETDT
241    NLNFQNLSVI GFRILLLKVA GFNLLMTLRL WSS
```

DAP12.PC tag

```
1      MVPCTLLLLL AAALAPTQTR AQAQSDSSCS TVSPGVLAGI VMGDLVLTVL IALAVYFLGR
61     LVPRGRGAAE AATRKQRITE TESPYQELQG QRSDVYSDLN TQRPYYKGSG EDQVDPRLID
121    GK
```

DAP12.HA tag

```
1      MVPCTLLLLL AAALAPTQTR AQAQSDSSCS TVSPGVLAGI VMGDLVLTVL IALAVYFLGR
61     LVPRGRGAAE AATRKQRITE TESPYQELQG QRSDVYSDLN TQRPYYKGSG YPYDVPDYA
```

DAP10.PC tag

```
1      MVPCTLLLLL AAALAPTQTR AQTTPGERSS LPAFYPGTSG SSSGCGSLSL PLLAGLVAAD
61     AVASLLIVGA VFLCARPRRS PAQEDGKVYI NMPGRGGSGE DQVDPRLIDG K
```

DAP10.HA tag

```
1      MVPCTLLLLL AAALAPTQTR AQTTPGERSS LPAFYPGTSG SSSGCGSLSL PLLAGLVAAD
61     AVASLLIVGA VFLCARPRRS PAQEDGKVYI NMPGRGGSGY PYDVPDYA
```

Fcγ.PC tag

```
1      MVPCTLLLLL AAALAPTQTR ALGEPQLCYI LDAILFLYGI VLTLLYCRLK IQVRKAAITS
61     YEKSDGVYTG LSTRNQETYE TLKHEKPPQG SGEDQVDPRL IDGK
```

Fcγ.HA tag

```
1      MVPCTLLLLL AAALAPTQTR ALGEPQLCYI LDAILFLYGI VLTLLYCRLK IQVRKAAITS
61     YEKSDGVYTG LSTRNQETYE TLKHEKPPQG SGYPYDVPDY D
```

CD3ε

```
1      MVPCTLLLLL AAALAPTQTR ADGNEEMGGI TQTPYKVSIS GTTVILTCPQ YPGSEILWQH
61     NDKNIGGDED DKNIGSDEDH LSLKEFSELE QSGYYVCYPR GSKPEDANFY LYLRARVCEN
121    CMEMDVMSVA TIVIVDICIT GGLLLLVYYW SKNRKAKAKP VTRGAGAGGR QRGQNKERPP
181    PVPNPDYEPI RKGQRDLYSG LNQRRI
```

CD3δ

```
1      MVPCTLLLLL AAALAPTQTR AFKIPIEELE DRVFVNCNTS ITWVEGTVGT LLSDITRLDL
61     GKRILDPRGI YRCNGTDIYK DKESTVQVHY RMCQSCVELD PATVAGIIVT DVIATLLLAL
121    GVFCFAGHET GRLSGAADTQ ALLRNDQVYQ PLRDRDDAQY SHLGGNWARN K
```

FuguDAP12.PC tag

```
  1     MVPCTLLLLL AAALAPTQTR ALFTACGHCY VIKTWVLMAV MITDILLTLL ITASVFCLMT
61     KLKRRRDPDR SEVTESPYQV LQGVQSDVYS ELETFSKGSG EDQVDPRLID GK
```

FuguDAP12.HA tag

```
  1     MVPCTLLLLL AAALAPTQTR ALFTACGHCY VIKTWVLMAV MITDILLTLL ITASVFCLMT
61     KLKRRRDPDR SEVTESPYQV LQGVQSDVYS ELETFSKGSG YPYDVPDYD
```

**B. Constructs for transfection experiments**

KIR2DS2.PC tag

```
1      MVPCTLLLLL AAALAPTQTR AEDQVDPRLI DGKHEGVHRK PSLLAHPGPL VKSEETVILQ
61     CWSDVRFEHF LLHREGKYKD TLHLIGEHHD GVSKANFSIG PMMQDLAGTY RCYGSVTHSP
121    YQLSAPSDPL DIVITGLYEK PSLSAQPGPT VLAGESVTLS CSSRSSYDMY HLSREGEAHE
181    RRFSAGPKVN GTFQADFPLG PATHGGTYRC FGSFRDSPYE WSNSSDPLLV SVTGNPSNSW
241    PSPTEPSSKT GNPRHLHVLI GTSVVKIPFT ILLFFLLHRW CSNKKNAAVM DQEPAGNRTV
301    NSEDSDEQDH QEVSYA
```

KIR2DS2-pVal.PC tag

```
1      MVPCTLLLLL AAALAPTQTR AEDQVDPRLI DGKHEGVHRK PSLLAHPGPL VKSEETVILQ
61     CWSDVRFEHF LLHREGKYKD TLHLIGEHHD GVSKANFSIG PMMQDLAGTY RCYGSVTHSP
121    YQLSAPSDPL DIVITGLYEK PSLSAQPGPT VLAGESVTLS CSSRSSYDMY HLSREGEAHE
181    RRFSAGPKVN GTFQADFPLG PATHGGTYRC FGSFRDSPYE WSNSSDPLLV SVTGNPSNSW
241    PSPTEPSSKT GNPRHLHVVV VVVVVKVVVV VVVVVVVHRW CSNKKNAAVM DQEPAGNRTV
301    NSEDSDEQDH QEVSYA
```

DAP12.HA tag

```
1      MVPCTLLLLL AAALAPTQTR AKYPYDVPDY AQAQSDSSCS TVSPGVLAGI VMGDLVLTVL
61     IALAVYFLGR LVPRGRGAAE AATRKQRITE TESPYQELQG QRSDVYSDLN TQRPYYK
```

TM Residues that were mutated as indicated in the text are underlined. Signal peptide is in green; tag sequences in red, linker peptide in blue and TM sequences in turquoise.
